# New Treatment Concepts in Implantology—The Role of Digital Innovations for an Individualized Dental Implant Rehabilitation

**DOI:** 10.3390/jcm11195667

**Published:** 2022-09-26

**Authors:** Lauren Bohner, Marcel Hanisch

**Affiliations:** Department of Cranio and Maxillofacial Surgery, Hospital University Münster, 48149 Münster, Germany

In the last few years, technological innovations have been changing the field of implantology. New treatment concepts and protocols have been offered for replacing missing teeth. In addition, advances in prosthodontic and surgical techniques, such as immediate loading, have broadened the treatment possibilities and increased the range of patients that may benefit from dental implant treatments.

Technological advancements have allowed acquisition of data to perform treatment planning based upon intraoral conditions, facial features and skeletal basis, thus providing accurate final outcomes. Accordingly, a virtual patient can be created through the integration of digital data acquired from extraoral, intraoral and tomographic scanners, such as photography devices. Hence, different treatment options can be virtually simulated, and discussed with the interdisciplinary team and with the patient. Based on these data, dental implantology plays a significant role in an interdisciplinary concept for oral–facial rehabilitation of the patient, on which not only the prosthetic plan, but also the soft tissue, facial tissue, and skeletal movements can be integrated to improve the final outcome.

Regarding the surgical planning, new technologies have been improving the diagnosis and detection of anatomical structures, increasing the reliability and safety of surgical procedures regardless of the complexity of the case. Digital devices, such as cone-beam computed tomography, allow three-dimensional (3D) visualization of anatomical structures, as well as quantitative and qualitative bone assessments. In addition, artificial intelligence has shown a great potential to facilitate and improve radiographic diagnosis. Thanks to the development of new software applications, different anatomical structures can be automatically segmented and visually assessed, saving time and facilitating the digital workflow.

Moreover, the possibility to integrate digital planning with surgical and prosthetic procedures by means of printing and developing a dental implant surface brings an option for a faster and more reliable dental implant rehabilitation. The optimization of dental material enables immediate dental implant placement and loading, considerably reducing treatment time and workflow and providing the same or even better outcomes than conventional protocols.

Hence, it must be recognized that many of these innovations are still under development and may have limitations. All advancements were only possible due to a broad scientific basis, and new studies are further required to ensure evidence-based treatments. In most cases, the basic background remains the same, and the knowledge of basic concepts is still essential to provide patients with optimal diagnosis and treatment. However, one thing is right: the future is changing. Dental implantology has become a segment of a personalized dental medicine, and, from now on, clinicians will be able to develop individualized dental implant rehabilitations for their patients.

